# Effects of the Incubation Period of *Pleurotus ostreatus* on the Chemical Composition and Nutrient Availability of Solid-State-Fermented Corn Stover

**DOI:** 10.3390/ani13162587

**Published:** 2023-08-11

**Authors:** Lydia K. Olagunju, Omoanghe S. Isikhuemhen, Peter A. Dele, Felicia N. Anike, Kelechi A. Ike, Yasmine Shaw, Rosetta M. Brice, Oluteru E. Orimaye, Michael Wuaku, Brandon G. Essick, Nathan Holt, Nkese S. Udombang, Judith O. Enemudo, Kiran Subedi, Uchenna Y. Anele

**Affiliations:** 1 Department of Animal Sciences, North Carolina Agricultural and Technical State University, 1601 East Market Street, Greensboro, NC 27411, USA; lkolagunju@aggies.ncat.edu (L.K.O.); delepa@funaab.edu.ng (P.A.D.); kaike@aggies.ncat.edu (K.A.I.); yashaw@aggies.ncat.edu (Y.S.); rosettab101@gmail.com (R.M.B.); oeorimaye@aggies.ncat.edu (O.E.O.); mwuaku@aggies.ncat.edu (M.W.); 2 Department of Natural Resources and Environmental Design, North Carolina Agricultural and Technical State University, 1601 East Market Street, Greensboro, NC 27411, USA; fnanike@ncat.edu (F.N.A.); bgessick@ncat.edu (B.G.E.); naholt@ncat.edu (N.H.); nsudombang@aggies.ncat.edu (N.S.U.); joenemudo@aggies.ncat.edu (J.O.E.); 3 Analytical Services Laboratory, North Carolina Agricultural and Technical State University, 1601 East Market Street, Greensboro, NC 27411, USA; ksubedi@ncat.edu

**Keywords:** *Pleurotus ostreatus*, inoculation period, dry matter digestibility, volatile fatty acid

## Abstract

**Simple Summary:**

White-rot fungi are known to selectively degrade lignin in biomass but were reported to require a longer fermentation period to complete the process. However, choosing an effective strain and knowing the optimum fermentation conditions could increase feeding quality and minimize nutrient loss in herbaceous biomass. The current study evaluated the effects of different periods of solid-state fermentation on the feed value and quality of corn stover. The results show that a 2 wk incubation period is sufficient to improve the nutritive value of *P. ostreatus*-treated corn stover.

**Abstract:**

The current study aimed to optimize and improve the feeding value of *Pleurotus ostreatus*-fermented corn stover by evaluating the effects of five solid-state fermentation times and three in vitro fermentation periods on the chemical composition, dry matter disappearance (DMD), microbial mass and volatile fatty acid (VFA) production of treated and untreated corn stover. The study utilized a 3 × 5 factorial design, with eight replicates per treatment. Dry matter, crude protein (CP), ash and non-fiber carbohydrate (NFC) contents increased quadratically (*p* < 0.05) with increases in the solid-state fermentation time. Increases of 44.4–59.1%, 20.6–78.6% and 40.5–121% were noted for the CP, ash and NFC contents, respectively. Organic matter, ether extract, neutral detergent fiber and hemicellulose contents decreased quadratically (*p* < 0.05) across the treatments. Similar trends were noted for DM and fiber disappearance in the treatments. The total gas production and in vitro true dry matter digestibility (IVTDMD) increased quadratically, while microbial mass and in vitro apparent DMD increased in a linear manner. The total VFA, propionate and butyrate contents increased linearly. Both the acetate content and the A:P ratio decreased in a linear manner. The results show that the rumen fermentation pathway favors the production of propionate, with increases in propionate production of 7.46 and 8.30% after 2 and 4 wk, respectively. The study showed that a 2 wk period of solid-state fermentation is sufficient to provide a bio-transformed cow–calf feed resource from *P. ostreatus*-treated corn stover.

## 1. Introduction

White-rot fungi (WRFs) degrade lignocellulosic materials efficiently via simultaneous attacks on lignin, cellulose, and hemicellulose due to their ability to release both hydrolytic and oxidative enzymes [1]. WRFs are described as the only fungi capable of complete lignin mineralization and can delignify lignocellulosic substrates selectively, thereby improving the nutritive value of these substrates for ruminants [2,3]. Selective lignin degraders have potential for the bioconversion of fibrous feed into cellulose-rich feed for animal production [4,5]. However, in ref. [3], it was reported that the composite nature of lignin degradation is regulated by enzyme synthesis, which might take a longer time when delignifying lignocellulosic substrates. 

The enzyme activity of WRFs has been noted to change considerably between the colonization and fructification stages [6]; thus, the synthesis and production of lignin-degrading enzymes are induced via limited nutrient levels in the secondary metabolism [2,7,8]. It was also reported that lignin degradation occurred faster during vegetative growth than fructification [9]. It was confirmed that the rate of degradation depends on the nutrients present, but it was also reported that the greater the amount of nutrients, the greater the degradation rate; however, the roles of some additional enzymes in lignin mineralization are significant [10]. An excessive dry matter loss of up to 30.9% is typical with WRFs after an extended or prolonged incubation period, so minimizing the incubation period to ensure a higher-quality final product was recommended [8].

A previous study [6] reported a weekly enzymatic cycle fluctuation in *Agaricus bisposrus*, but other reports [7,10] highlighted that a longer period is required for the complete mineralization of lignocellulosic substrates. In ref. [11], it was reported that despite positive responses regarding the digestibility of the treated substrates, the limited use of WRFs in ruminant nutrition is partly due to the extended pretreatment time required. Consequently, in ref. [12], it was recommended that optimization studies are required to shorten the process of delignification. Similarly, the author of ref. [2] noted the need for additional research to achieve a roughage–fungi mixture of optimal quality. In ref. [13], it was reported that the prospect of utilizing unexploited fibrous feed is significant if value-added products can be generated through an environmentally friendly method/process. Hence, studies to optimize the fermentation period for lignin removal by WRFs are important for enhancing the conversion of lignocellulosic biomass into quality feed. Corn stover is widely distributed, abundant (contributing about 35% of the global annual straw production) and a cheap source of energy for ruminants with a great potential for development and utilization [14]. Therefore, measures to improve its digestibility are important to provide cheaper feed resources. We chose *Pleurotus ostreatus*, which produces edible mushrooms; it is a selective WRF that is already used in several biotechnological applications [13]. Also, it is reported that the ability of WRFs to degrade a structural carbohydrate is dependent on the substrate and the fermentation time [15]. In the current study, the hypothesis is that incubation time for solid-state fermentation by *P. ostreatus* will significantly affect the delignification and feed value of corn stover. The specific objectives were to evaluate the effects of incubation time on dry matter disappearance, fiber digestibility, the partitioning factor, microbial mass, volatile fatty acid concentrations and the energy values of *P. ostreatus*-treated corn stover.

## 2. Materials and Methods

### 2.1. Spent Mushroom Substrate Cultivation and Processing

Spent mushroom substrates were processed as previously reported in [16], but instead of a single fermentation period of 3 weeks, the substrates were fermented for 0, 2, 4, 6 and 8 weeks. The corn stover was shredded using chromium chipper blades and a gas-powered 205 cc CS 4265 TROY-BILT chipper shredder (Model 24B-412J711) with an 800 series Briggs & Stratton engine and a 2-inch chipping capacity. Thereafter, the chips were packed into bags (each weighing 2 kg) and soaked in water for 18 h using a 55-gallon drum. The drum was inverted and allowed to drain for 12 h. The bags were sterilized in an autoclave for 1 h and left inside the autoclave for 13 h (overnight) to cool down. The bags were transferred from the autoclave to a laminar flow hood and inoculated with 5% (wet weight) *P. ostreatus* grain spawn. After inoculation, the bags were sealed using an impulse sealer and transferred to the incubation room, where the mycelial colonization of the substrate block (spawn-run) occurred over the different fermentation periods listed above. All other conditions were as described in [16].

### 2.2. Animal Care and Feeding

Rumen fluid was sampled from dairy cows maintained under an IACUC-approved protocol, LA21-009.

### 2.3. Experimental Design

The experiment employed a 3 × 5 factorial design with two separate runs. The treatments were 0 (untreated, used as a control), 2, 4, 6 and 8 wk of treating corn stover with *P. ostreatus*. The treatments were evaluated for nutrient digestibility at 6, 24 and 48 h time periods via the in vitro batch culture technique. For each run and at each incubation period, four replicates were prepared for each treatment (eight replicates for the study).

### 2.4. Sample Preparation

The samples were prepared as previously reported in [16]. Half a gram of each spent mushroom substrate was weighed into an Ankom F57 bag and sealed using an MP-8 Intertek heat sealer. The bags were then inserted into pre-labeled 100 mL serum bottles that were arranged on a stainless-steel tray and placed inside an incubator.

### 2.5. In Vitro Batch Culture

A detailed description of the batch culture study was previously reported in [16,17]. The inoculum was sampled from two ruminally cannulated Holstein cows. The animals were fed the same diet as previously reported, and they had unrestricted access to clean drinking water. The inoculum was mixed with artificial saliva [18] in a 1:3 ratio (15 mL of inoculum: 45 mL of artificial saliva) and incubated at 39 °C for 6, 24 and 48 h. Using a pressure transducer, the accumulated headspace gas pressure was measured at each of the three times points. Blanks were included to correct for the production of gas from the buffered inoculum. Corrected gas pressure values were used to estimate the gas production [19]. 

### 2.6. Laboratory Analysis

#### 2.6.1. Chemical Analysis

The ash content of the samples was measured via the procedure described in [20]. The organic matter (OM) was calculated by subtracting the ash content from 100. The samples were analyzed for their nitrogen content via the Pregl–Dumas method. The crude protein (CP) content was estimated by multiplying the nitrogen value by 6.25. Petroleum ether was used to determine the ether extract (EE) content [21], using an Ankom XT15 fat analysis system (Ankom Technology Corp., Fairport, NY, USA). The non-fibrous carbohydrate (NFC) content was calculated by subtracting the sum of the ash, CP, neutral detergent fiber (NDF) and EE contents from 100, according to [22]. Subsamples of the treatments and incubation residues were dried in a forced-air oven at 55 °C for 48 h to estimate the DM. The procedure described in [22] was used to analyze the NDF content, and the acid detergent fiber (ADF; [23]) content was analyzed sequentially, using the same bags as the NDF analysis. The acid detergent lignin (ADL) content was analyzed using 72% sulfuric acid (H_2_SO_4_). The hemicellulose content was calculated using NDF-ADF, and the cellulose content was calculated using ADF-ADL.

#### 2.6.2. Microbial Mass

The microbial mass was determined according to the protocol described in [24], with a slight modification. The pellet samples were removed from the freezer and decapped before they were arranged in aluminum pans. The samples were lyophilized for 96 h using a BUCHI freeze-dryer (model DUO 6 M; BUCHI Labortechnik AG, Flawil, Switzerland). The lyophilized samples were weighed to determine the weight of the pellets, and the microbial mass was calculated as reported in [24]. The partitioning factor (PF) was estimated as the ratio of mg of substrate truly degraded/mL of gas produced, according to the procedure in [25].

#### 2.6.3. Volatile Fatty Acid

The preserved rumen fluid samples were thawed and centrifuged at 10,000× *g* for 15 min at 4 °C, and the volatile fatty acid (VFA) concentration was analyzed as described in [26] (2020). Gas chromatography [16] with Flame Ionization Detection (FID) was used to quantify the VFA concentration, and a metaphosphoric–crotonic acid mixture was used as an internal standard. The sample injection volume was set at 1 μL while maintaining a split ratio of 1:12. The injector port was kept at a constant temperature of 250 °C. Helium was used as the carrier gas at a flow rate of 1 mL/min, facilitating the efficient transport of the sample through the GC column. A temperature gradient was employed in the oven to optimize the separation of the analytes. Initially, the oven temperature was set at 120 °C for 0.8 min, followed by a controlled increase of 8 degrees per minute until 140 °C was reached. The oven was maintained at 140 °C for 1.8 min. The detector temperature was maintained at 280 °C. The FID operation was supported by a controlled flow of hydrogen and air gases with flow rates of 30 mL/minute and 400 mL/minute, respectively. Additionally, N was used as a make-up gas at a flow rate of 25 mL/min, ensuring a stable baseline and consistent detector performance.

#### 2.6.4. Estimated Parameters

The estimated parameters were calculated using the following formulae: dry matter intake (DMI) = 120/NDF; dry matter digestibility (DDM) = 88.9 − (0.779 × ADF); relative forage value (RFV) = (DDM × DMI) × 0.775; relative forage quality (RFQ) = (DMI × TDN)/1.23; total digestible nutrient (TDN) = 104.97 − (1.302 × ADF); digestible crude protein (DCP) = (0.916 × CP) − 3.09; gross energy (GE) = (CP × 0.056) + (EE × 0.094) + (100 − CP − Ash − EE) × 0.042; digestible energy (DE) = TDN × 0.04409; metabolizable energy (ME) = 0.821 × DE; net energy of maintenance (NEM) = (TDN × 0.029) − 0.29; net energy of gain (NEG) = (TDN × 0.029) − 1.01; net energy of lactation (NEL) = (TDN × 0.0245) − 0.012 [27,28,29,30,31].

### 2.7. Statistical Analysis

The data generated were analyzed using the MIXED procedure in SAS software (Version 9.4) [32] (SAS Institute Inc., Cary, NC, USA). The solid-state fermentation period, in vitro batch culture incubation time and their interactions were treated as fixed effects, and the run was treated as a random effect. Significance was declared at *p* < 0.05, and a trend was considered for 0.05 ≤ *p* ≥ 0.10 unless otherwise stated.

Data were analyzed using the model shown below:

Y_ijk_ = µ + SSF_i_ + T_j_ + (SSF × T)_ij_ + e_ijk_

where Y_ijk_ is the dependent variable, µ is the overall mean, SSF_i_ is the solid-state fermentation effect, T_k_ is the batch culture incubation time effect and e_ijk_ is the residual error. Orthogonal polynomial contrasts were used to detect trends (linear and quadratic) among the solid-state fermentation treatments.

## 3. Results and Discussion

### 3.1. Chemical Composition

Compared to the control treatment (a 0 wk fermentation period), the dry matter increased quadratically (*p* < 0.001), increasing from 5.69 to 6.78% across the treatments (Table 1). The increase in DM content due to the solid-state fermentation of the corn stover by *P. ostreatus* could result in more nutrients in the treated samples. Dry matter contains all the nutrients that will be available to the animals, and it plays an important role in animal nutrition as rations are formulated on a DM basis. Mycelia are noted to secrete enzymes to degrade, solubilize and release nutrients bound in lignocellulosic substrates before transporting them to developing fungal primordia and fruiting bodies [33]; this could have resulted in the increased accumulation of nutrients in wk 2, 4, 6 and 8 of the corn stover substrate treatment.

By altering the chemical nature of the substrates, both the CP and ash concentrations were quadratically increased (*p* < 0.05) in the present study, ranging from 44.4–59.1% and 20.6–78.6%, respectively. Although the increases in ash content for the wk 2, 4 and 6 treatments were within the recommended value for total mixed rations and forage [34], there were reports [4,35] of a negative effect on voluntary intake when animals were fed diets containing high concentrations of ash. The increase in the CP concentration could be due to the presence of amino acids from the fungi (secondary metabolites), which could increase the presence of amino acids in the treated substrates. Previous reports indicate that some metabolites were generated during the bioconversion process, and the production of nitrogen-containing secondary metabolites was confirmed in higher fungi [36,37]. Therefore, supplementing *P. ostreatus*-treated substrates could provide additional amino acids to meet the nutrient requirements of ruminants, which could increase the efficiency of animal production with a low-cost ration formulation. The non-linear patterns noted in the present study could have resulted from the twin peaks in enzyme production, as reported previously [8]. The trend noted for CP across the fermentation periods was inconsistent with a previous study [38], which reported that the CP content increased with increases in the incubation period. 

The ether extract content decreased quadratically (*p* = 0.006) across the treatments, decreasing by 55.3%, 52.1%, 47.9% and 42.3% in wk 2, 4, 6 and 8 of the treatments, respectively. The decrease in the EE content could have resulted from the presence of mevinolin (HMG-CoA reductase inhibitor) in *P. ostreatus*, which is known to have a lipid-lowering effect [39]. The presence of this enzyme could provide a hypocholesterolemic feed ingredient for animals to decrease low-density lipoprotein in animal products. The lowest EE content noted for wk 2 could be related to the highest CP content noted for the sample, and this observation is consistent with a previous study [8,40], which reported that microorganisms convert carbon into proteins through an intermediate metabolism. Therefore, the decrease in the EE content was speculated to have been used for amino acid synthesis, resulting in the greatest increase in the CP content for the corn stover treated for 2 wk.

Additionally, the lower EE content noted in the corn stover fermented for 2 wk tends to favor the activities of rumen microbes [41,42] as a higher EE concentration is known to have negative effect on the digestibility of DM. Thus, the 2 wk treated corn stover demonstrated a better nutrient profile in terms of DM, CP, ash and EE. Several reports [1,7,43] noted that substrates with high carbon sources and lower nitrogen contents are essential for the optimum growth of *Pleurotus* spp. Likewise, in [13], it was reported that a low hemicellulose content combined with a high lignin content induced the activities of relevant enzymes. Therefore, the effect of enzymatic activities on the chemical composition of the corn stover across the fermentation periods is consistent with a previous study [44], which reported continuous changes in *Pleurotus* spp.-fermented cereal straw. Hence, the data from the current study show that changes in the substrate’s chemical composition are dependent on the fermentation period [45]. 

### 3.2. Loss of Organic Matter

The organic matter content decreased quadratically (*p* < 0.05) from wk 2 to wk 6 before demonstrating a slight increase in wk 8 of the treatment. The decreases in OM were 1.14, 0.831 and 3.0% for wk 2, 4 and 6, respectively, before a significant increase in wk 8 of the treatment. The reason for this observation is inconclusive at this time, although one could speculate that an increase in fungal biomass as the fermentation period increases could be responsible. The decrease in OM in wk 2, 4 and 6 could have resulted from the utilization of minerals from the cellulolytic constituents by *P. ostreatus* for its growth without a corresponding increase in biomass accumulation. Nevertheless, the decrease in OM is consistent with previous studies [37,43,44,46,47], although it was less pronounced in the current study. Consistent with the current results, in [48], a gradual decrease in biomass loss with an increase in enzyme production was reported, and it was also noted that only oxidative enzymes would influence biomass loss, which explains why the loss of OM was reversed at 8 weeks. The results from the current study are contrary to the results in [47], in which it was reported that a significant loss of OM is a major limitation in using *P. ostreatus* as a loss of OM is considered inefficient [44].

### 3.3. Fiber Fractions

The neutral detergent fiber and hemicellulose contents decreased quadratically (*p* < 0.001) across the treatments (Table 1). A contrasting observation was noted for the ADF content, which decreased linearly (*p* < 0.001) from wk 0 to wk 4. Compared to the control treatment, higher ADF contents were observed for the wk 6 and 8 treatments. Lower ADF and NDF values at wk 2 and 4 indicate better nutritive value for these two treatments, which would increase digestibility and the availability of nutrients to the animals. This is consistent with a previous study [35] which reported decreases in ADF and NDF contents in *P. ostreatus*-treated rice straw. It was also reported that non-linear changes in ADF and NDF contents were influenced by the incubation time [49].

Isikhuemhen and Mikiashvilli [6,40] reported that the ligninolytic enzyme activity of *P. ostreatus* during the growth period of mycelial (the vegetative phase) colonization was higher and declined during the development of fruiting bodies (the reproductive phase), which was speculated to begin approximately 4 weeks after inoculation. These reports are consistent with results noted for the wk 2 and 4 samples. Melanouri [7] also reported that complete colonization by *P. ostreatus* occurs between 16 and 36 days. Furthermore, Julian [1] reported that extensive enzymes are produced during the vegetative phase to biodegrade lignocellulosic materials; then, the dissolved nutrients are used to support fruiting body formation and development. Zadrazil [44] also reported that *Pleurotus* spp. metabolized readily soluble components of the substrate for the production of its own biomass during substrate colonization. This is also consistent with previous reports that the mycelium releases enzymes which diffuse into the substrate to hydrolyze polysaccharides and consume soluble sugars [50,51]. The significant (*p* < 0.001) reduction in the hemicellulose content at wk 2 and 4 could have resulted from the increasing demand for additional energy as mycelial growth progressed, which can also be attributed to the variation in lignin degradation. The variation in the ADL content resulted in a numerical increase (*p* > 0.05) between the wk 2 sample and the control, with a further numerical decrease for the wk 4 sample, which is consistent with the results of two previous studies [33,44] which reported that lignin is degraded during the second phase.

It was observed that hemicellulose was preferentially degraded compared to cellulose, and this observation is consistent with previous studies [6,43] which reported that hemicellulose is required before efficient lignin degradation can commence. Likewise, in Ref. [52], a low level of cellulase activity during lignocellulose-based substrate degradation was reported, but it is very important for the completion of the degradation process. For the extensive degradation of lignin with minimum cellulose utilization, it is desirable to ensure that an adequate amount of the nutrient is retained for animal production [8]. The current study indicated minimum cellulose utilization with respect to lignin degradation, thereby causing 9.89 and 5.62% reductions in cellulose for wk 2 and 4, respectively. The non-fiber carbohydrate content increased quadratically (*p* < 0.05), increasing from 40.5 to 121% across the treatments. The increase in the NFC content was more pronounced during the preliminary stage of fermentation, with increases of 116% and 121% for the wk 2 and 4 samples, respectively. Therefore, it is speculated that the digestibility of the treated corn stover after wk 2 and 4 would be rapid, with a faster rate of passage and a shorter residence time.

Greater (quadratic effect, *p* < 0.05) DM digestibility (DMD), ADF digestibility (ADFD), ADL digestibility (ADLD) and cellulose digestibility (CELLD) were observed with the wk 2, 4, 6 and 8 treatments compared with the control treatment (Table 2). The neutral detergent fiber digestibility (NDFD) and hemicellulose digestibility (HEMD) were decreased (*p* < 0.001) linearly and quadratically, respectively. Consistent with the trend noted in the chemical composition results, the wk 2 and 4 samples had the highest (quadratic effect, *p* < 0.001) DMD values after 48 h of in vitro batch culture fermentation. The increase in DMD showed that the treated substrates would be digested better and had more utilizable nutrients than the control, with the wk 2 and 4 samples having the best digestibility due to delignification by *P. ostreatus*.

The results from the current study show that a shorter solid-state fermentation time is optimum for the biological delignification of corn stover to produce quality feed material. This is consistent with a previous study [48], which reported that the maximum specific activities of *P. ostreatus* were obtained between 9 and 20 days (2 wk) and confirmed that high levels of ligninolytic enzymes can be produced within the first few days of solid-state fermentation. Contrary to the current study, in [38], a linear, consistent increase in digestibility was reported for all fermentation periods. Similarly, in [44], a lower degree of digestibility was reported in the early phase of fermentation (30 days, which corresponds to 4 wk), and that degree of digestibility increased as the fermentation continued from d 60 to 105. Contrary to these differences, the authors reported a similar digestibility value at 15 days, which is consistent with the value from the current study (at 2 wk).

With the exception of ADLD, the other fiber fractions had higher (*p* < 0.001) digestibility values at either 6 h (NDFD, ADFD, HEMD and CELLD) or 24 h (ADFD and CELLD). Lower (linear effect, *p* < 0.001) NDF and hemicellulose contents in the wk 2 and 4 samples also resulted in lower NDFD and HEMD values across the three in vitro batch culture incubation periods. Similarly, higher (quadratic effect, *p* < 0.001) NDF and hemicellulose values for the control treatment resulted in the highest NDFD and HEMD values for the control after 6 h of in vitro batch culture fermentation. The extensive disappearance of NDF and hemicellulose at 6 h in the control indicated that some digestible portions of the substrates were utilized during solid-state fermentation [44]. 

### 3.4. In Vitro Gas Production and the Efficiency of Microbial Protein Synthesis

The total gas production and in vitro true dry matter digestibility (IVTDMD) increased (*p* < 0.05) quadratically, while the microbial mass and in vitro apparent DMD (IVADMD) increased (*p* < 0.001) in a linear manner (Table 3). The partitioning factor and the undegraded portion of the treatments decreased (*p* < 0.05) quadratically, with greater values observed after 6 h of in vitro batch culture fermentation. The highest gas production was noted for the wk 2 and 4 treatments after 24 h of in vitro batch culture fermentation. Differences in gas production reflected variations in biological delignification which could have resulted from the extent to which the transformation of modifying the cell wall into readily fermentable fiber had occurred in each incubation period. Hence, the greater amount of gas produced in the treated samples (wk 2, 4, 6 and 8) could be the result of access to fermentable carbohydrates [37]. The greater gas production noted for the wk 2 and 4 samples agrees with this assumption, as their NFC concentrations increased by 116% and 121%, respectively. It is evident that more nutrients would be available to the animals for use by the rumen microbes, which indicates better nutritive value for the wk 2 and 4 treated corn stover. 

The results show that the ligninolytic enzymatic activities of *P. ostreatus* had a positive effect on the lignin complex of the substrates, which bio-transformed the non-structural carbohydrate concentration, resulting in higher values for the wk 2 and 4 samples. This would ensure the availability of energy for microbial growth and invariably microbial protein synthesis as plant components have the potential to modulate rumen fermentation toward maximizing the synthesis of the microbial biomass. Compared with the control sample, the MM increased (*p* < 0.001) linearly by 100–755%, 112–627%, 35.3–330% and 22.6–355% for wk 2, 4, 6 and 8, respectively. The observed increase could be attributed to a balanced supply of CP, fungal-secreted polysaccharides and the energy in the treated samples since nutrient deficiency in the rumen will not allow rumen microbes to grow efficiently. Thus, the outstanding results noted for the wk 2 and 4 samples indicate their potential for efficient microbial protein synthesis to improve animal performance. Microbial protein is an excellent alternative source of protein for ruminants, and a high degree of carbon fixation to microbial cells can reduce fermentable carbon losses in the form of gases, especially carbon dioxide and methane [25]. Bowen [53] reported that increasing fiber concentration resulted in a linear decrease in the efficiency of microbial protein synthesis, which was evident in the wk 6 and 8 samples. 

Overall, the results from the present study are consistent with a previous study [53] which noted that structural and non-structural carbohydrates can influence the efficiency of microbial protein synthesis (EMPS) through their effects on microbial maintenance requirements in the rumen. The highest (quadratic effect, *p* < 0.001) IVTDMD values were noted after 48 h of in vitro batch culture fermentation, with increases of 30.5–111% and 29.7–108% in the wk 2 and 4 samples, respectively, compared with increases of 8.53–59.4% and 8.92–61.7% in the wk 6 and 8 samples, respectively. The same trend was noted for IVADMD, with the wk 2 and 4 samples having the highest (linear effect, *p* < 0.001) values after 48 h of in vitro batch culture fermentation. The increase in DM digestibility also resulted in lower (quadratic effect, *p* < 0.001) undegraded portions noted for the wk 2 and 4 samples at 48 h, indicating that a considerable portion of the feed would be digested in the wk 2 and 4 samples and would provide more nutrients to the animal compared to the control. The fermentation by *P. ostreatus* quadratically decreased (*p* = 0.003) the PF of the treated corn stover compared to the control. We expected better PF values from the treated samples due to their higher MM values, but the PF values were estimated based on the ratio of truly degraded substrate per gas produced, and higher total gas production in the treated samples was responsible for the lower PF values noted in the current study. Contrary to some previous studies [41,54], which reported an inverse relationship between in vitro gas production and microbial biomass yield, there was a linear relationship between these two variables. This could be due to the delignification process, which resulted in a significant increase in NFC content in the treated group when compared with the control. 

### 3.5. Volatile Fatty Acid

The total VFA, propionate and butyrate contents increased linearly (*p* < 0.05; Table 4). Both the acetate content and A:P ratio decreased (*p* < 0.05) in a linear manner. No treatment effect was observed for the concentrations of iso-butyrate, valerate and iso-valerate. The values for TVFA, propionate, butyrate and iso-butyrate were the highest (linear effect, *p* < 0.05) for the wk 2 and 4 samples after 48 h of in vitro batch culture incubation. The improved digestibility of the treated samples influenced the products of fermentation as structural carbohydrates and NFC contents differed with the rate and extent of fermentation. It could be summarized that the high proportions of NFC content in the wk 2 and 4 samples were responsible for the observed TVFA values. This result is consistent with a previous study [12] which reported that rumen microbes will degrade more substrates when more carbohydrates are available, resulting in an increase in the production of VFA. A significant decrease (*p* < 0.001) in the production of acetate in the wk 2 and 4 samples when compared with the control showed a shift in the rumen fermentation pathway because of the increased amount of non-structural carbohydrates in the two treated samples. The results show that the rumen fermentation pathway favored the production of propionate, with increases of 7.46% and 8.30% in propionate production after 2 and 4 wk, respectively. This shift in the fermentation pathway could be detrimental to the activities of methanogenic bacteria and could result in reduced methane production and energy loss in ruminants. The fermentation pathways noted for the wk 6 and 8 samples indicated the presence of more structural carbohydrates when compared with the wk 2 and 4 samples. 

Differences in the fermentation pathways for the wk 2, 4, 6 and 8 samples showed the effects of the fermentation time on the biological delignification of the *P. ostreatus*-treated corn stover. The current results provide evidence that the digestibility of the wk 2 and 4 samples was better and could occur faster as the rate of substrate degradation in the rumen determines the amount of energy that becomes available for rumen microbes since propionate is associated with a fast rate of substrate degradability in the rumen. Additionally, the wk 2 sample demonstrated a greater potential for increased butyrate production, with the highest value of 0.089 at 48 h, which could be beneficial for the papillae development in calves. The results show that 2 weeks of fermentation is sufficient for the biological treatment of corn stover with *P. ostreatus,* and this observation is consistent with previous studies [48,49], ref. [48], which reported that 2 weeks is sufficient for the maximum ligninolytic enzymatic activity of *P*. *ostreatus.*

### 3.6. Estimated Parameters

The dry matter intake, TDN, DDM and DCP increased quadratically (*p* < 0.05) across the treatments (Table 5). Linear increases (*p* < 0.001) were observed for the RFV and RFQ. Compared with the control treatment, a quadratic effect was observed for the GE, with values decreasing by 6.14%, 5.48%, 7.23% and 3.72% for the wk 2, 4, 6 and 8 treatments. All the remaining energy estimates increased quadratically (*p* = 0.016) across the treatments. With the exception of the GE, the other estimated parameters were higher (linear (*p* < 0.001) and quadratic (*p* < 0.05) effects) for the wk 2 and 4 treatments compared to the wk 6, 8 and control treatments. The increased biological delignification processes in the wk 2 and 4 samples could be responsible for this observation. Higher levels of digestibility and DMI are essential in improving production performance in any livestock sector [25]. Th results show that the solid-state fermentation of corn stover with *P. ostreatus* for 2–4 weeks could increase the utilization of the corn in livestock production by increasing the DMI of corn stover, which is typically known to have a lower DMI value due to its lignified cell walls [55]. Additionally, the decreases in the NDF and ADF contents of these two samples could have resulted in the increase in the DMI, and this is consistent with previous studies [4,56] that reported an increase in the daily DM consumption of fungi-treated straw in an in situ study.

The lower GE values in the treated samples might have resulted from the utilization of OM in the treated groups for fungal growth [4,43]. Also, the small loss in OM slightly increased the ash content in the treated samples, which might have resulted in lower GE values in the treated groups. This assumption is consistent with the results in [57], who reported that GE increases as ash concentration decreases. It is also good to note that the GE content does not indicate the availability of energy to the animals, and the other energy values were better in the treated samples. For example, the digestible energy, estimated metabolizable energy, net energy of maintenance, net energy gain, and net energy of lactation, which were higher in the treated samples, provide better information on energy availability in the rumen and are important for predicting microbial protein synthesis; this is consistent with the higher MM yield noted for the treated samples in the current study.

## 4. Conclusions

Based on our results, 2 weeks is sufficient for the biological treatment of corn stover with *P*. *ostreatus* to provide a nutrient-enhanced material that is useful as a supplement feed resource in feeding ruminants. The results show that a long fermentation period is not required to achieve the delignification and optimal quality of *P. ostreatus*-treated corn stover. Biodegradation or biomodification by *P. ostreatus* is a complex process associated with mycelial growth and the extracellular enzyme production system. Further studies are recommended at the farm level to evaluate animal performance and the levels of inclusion of the substrate. In addition, in vitro studies to understand enzyme production and dynamics and amino acid synthesis during the solid-state fermentation of corn stover with *P. ostreatus* are recommended.

## Figures and Tables

**Table 1:** Effect of inoculation period on chemical composition of treated and untreated corn stover.

Variable	Fermentation Period, wk						Contrasts (*p*-Values)	
	0	2	4	6	8	SEM	Linear	Quadratic
DM, %	91.4	97.6	97.2	96.6	96.8	0.53	<0.001	<0.001
OM, %	96.3	95.2	95.5	93.4	96.5	0.27	<0.001	0.043
CP, %	3.42	5.44	5.41	4.94	5.14	0.216	<0.001	<0.001
ASH, %	3.69	4.82	4.45	6.59	3.49	0.270	<0.001	0.043
EE, %	8.92	3.99	4.27	4.64	5.15	0.484	<0.001	0.006
NFC, %	12.6	27.2	27.9	17.7	21.1	1.35	<0.001	<0.001
NDF, %	71.3	59.6	58.0	66.1	65.1	1.16	<0.001	<0.001
ADF, %	44.7	41.2	42.2	46.5	46.6	0.56	0.001	0.016
ADL, %	7.24	7.50	6.84	9.29	7.19	0.269	0.090	0.705
HEM, %	26.7	17.4	15.8	19.6	18.5	0.87	<0.001	<0.001
CELL, %	37.4	33.7	35.3	37.2	39.4	0.53	<0.001	0.067

DM, dry matter; OM, organic matter; CP, crude protein; EE, ether extract; NFC, non-fiber carbohydrate; NDF, neutral detergent fiber, ADF, acid detergent fiber; ADL, acid detergent lignin; HEM, hemicellulose; CELL, cellulose.

**Table 2:** Effect of inoculation period on dry matter and fiber disappearance of treated and untreated corn stover.

Time, h	Trt, wk	DMD, %	NDFD, %	ADFD, %	ADLD, %	HEMD, %	CELLD, %
6	0	5.03 ^j^	86.2 ^a^	61.5 ^f^	13.5 ^h^	24.8 ^a^	48.0 ^cdef^
	2	17.0 ^h^	74.8 ^d^	69.0 ^cd^	15.5 ^efgh^	5.79 ^h^	53.5 ^ab^
	4	17.2 ^h^	75.6 ^d^	71.3 ^a^	15.3 ^fgh^	4.25 ^h^	56.0 ^a^
	6	13.3 ^i^	82.2 ^b^	68.8 ^cd^	17.5 ^defg^	13.4 ^d^	51.3 ^bc^
	8	13.5 ^i^	81.8 ^b^	69.9 ^bc^	14.5 ^gh^	11.9 ^de^	55.4 ^a^
24	0	20.5 ^g^	82.9 ^b^	62.05 ^f^	14.6 ^gh^	20.8 ^b^	47.4 ^def^
	2	29.3 ^de^	78.0 ^c^	69.9 ^bc^	18.8 ^cde^	8.16 ^g^	51.1 ^bcd^
	4	30.7 ^d^	79.0 ^c^	70.6 ^ab^	18.1 ^def^	8.45 ^g^	52.5 ^ab^
	6	23.6 ^f^	82.4 ^b^	68.7 ^cd^	19.9 ^cd^	13.8 ^d^	48.7 ^cde^
	8	28.0 ^e^	81.8 ^b^	68.0 ^d^	17.2 ^defg^	13.7 ^d^	50.8 ^bcd^
48	0	37.3 ^c^	82.5 ^b^	60.9 ^f^	17.8 ^defg^	21.7 ^b^	43.1 ^gh^
	2	52.4 ^a^	77.8 ^c^	68.3 ^d^	23.8 ^ab^	9.52 ^fg^	44.5 ^fgh^
	4	52.7 ^a^	79.1 ^c^	68.3 ^d^	21.8 ^bc^	10.8 ^ef^	46.5 ^efg^
	6	42.0 ^b^	81.7 ^b^	67.6 ^d^	25.5 ^a^	14.1 ^cd^	42.2 ^h^
	8	42.1 ^b^	82.0 ^b^	66.0 ^e^	17.8 ^defg^	16.0 ^c^	48.2 ^cde^
SEM		1.306	0.30	0.31	0.40	0.546	0.47
*p*-values							
Time		<0.001	0.084	<0.001	<0.001	<0.001	<0.001
Linear		<0.001	<0.001	<0.001	<0.001	<0.001	0.171
Quadratic		<0.001	0.117	<0.001	0.033	<0.001	<0.001
Trt × Time		<0.001	<0.001	<0.001	<0.001	<0.001	<0.001

Trt, treatment; DMD, dry matter digestibility; NDFD, neutral detergent fiber digestibility; ADFD, acid detergent digestibility; ADLD, acid detergent lignin digestibility; HEMD, hemicellulose digestibility; CELLD, cellulose digestibility. ^a–h^ Means with different superscripts within each column differ by *p* < 0.05.

**Table 3:** Effect of inoculation period on gas production, in vitro digestibility, and efficiency of microbial production for treated and untreated corn stover.

Time, h	Trt, wk	Gas, mL	PF	MM, g/kg DM	Undegraded	IVADMD	IVTDMD
6	0	3.56 ^g^	11.6 ^a^	0.011 ^i^	0.39 ^a^	0.18 ^f^	0.18 ^i^
	2	14.5 ^g^	10.4 ^b^	0.094 ^a^	0.32 ^c^	0.20 ^f^	0.38 ^f^
	4	12.7 ^g^	9.76 ^bc^	0.080 ^ab^	0.31 ^c^	0.22 ^f^	0.37 ^f^
	6	11.0 ^g^	9.67 ^bc^	0.044 ^def^	0.36 ^b^	0.20 ^f^	0.29 ^h^
	8	10.4 ^g^	8.89 ^c^	0.050 ^cde^	0.35 ^b^	0.19 ^f^	0.29 ^h^
24	0	40.2 ^f^	2.69 ^d^	0.017 ^hi^	0.31 ^cd^	0.32 ^e^	0.34 ^g^
	2	64.5 ^d^	2.33 ^de^	0.034 ^efgh^	0.28 ^e^	0.38 ^d^	0.45 ^d^
	4	62.1 ^d^	2.40 ^de^	0.036 ^efgh^	0.28 ^e^	0.38 ^d^	0.45 ^d^
	6	46.4 ^ef^	2.36 ^de^	0.023 ^ghi^	0.31 ^c^	0.34 ^e^	0.37 ^f^
	8	57.5 ^de^	2.57 ^d^	0.043 ^defg^	0.30 ^cd^	0.34 ^e^	0.41 ^e^
48	0	113 ^c^	1.52 ^e^	0.031 ^efghi^	0.25 ^f^	0.43 ^c^	0.48 ^c^
	2	189 ^a^	1.41 ^e^	0.062 ^bcd^	0.19 ^g^	0.50 ^a^	0.63 ^a^
	4	186 ^a^	1.37 ^e^	0.066 ^bc^	0.19 ^g^	0.49 ^a^	0.63 ^a^
	6	132 ^b^	1.51 ^e^	0.027 ^fghi^	0.24 ^f^	0.48 ^ab^	0.53 ^b^
	8	137 ^b^	1.43 ^e^	0.038 ^efgh^	0.24 ^f^	0.45 ^bc^	0.53 ^b^
SEM		5.755	0.363	0.0026	0.005	0.011	0.011
*p*-values							
Time		<0.001	<0.001	<0.001	<0.001	<0.001	<0.001
Linear		<0.001	0.134	<0.001	<0.001	<0.001	<0.001
Quadratic		0.015	0.003	0.090	0.008	0.053	<0.001
Trt × Time		<0.001	<0.001	<0.001	<0.001	<0.001	<0.001

Trt, treatment; PF, partitioning factor; MM, microbial mass; IVADMD, in vitro apparent dry matter digestibility; IVTDMD, in vitro true dry matter digestibility. ^a–i^ Means with different superscripts within each column differ by *p* < 0.05.

**Table 4:** Effect of inoculation period on total and individual volatile fatty acid production for treated and untreated corn stover.

Time, h	Trt, wk	TVFA, mmol	Acetate	Propionate	Butyrate	Iso-Butyrate (10^−3^)	Valerate (10^−3^)	Iso-Valerate (10^−3^)	A:P Ratio
6	0	29.5 ^e^	0.739 ^ab^	0.183 ^fg^	0.069 ^e^	2.03 ^abcd^	2.69	3.47	4.09 ^ab^
	2	33.6 ^e^	0.724 ^c^	0.194 ^def^	0.075 ^bcde^	1.62 ^cd^	2.57	3.29	3.76 ^cd^
	4	33.1 ^e^	0.728 ^bc^	0.191 ^ef^	0.073 ^cde^	1.74 ^bcd^	2.58	3.26	3.84 ^bcd^
	6	33.3 ^e^	0.748 ^a^	0.175 ^g^	0.069 ^e^	1.87 ^abcd^	2.55	2.89	4.31 ^a^
	8	31.3 ^e^	0.733 ^bc^	0.187 ^fg^	0.072 ^cde^	1.74 ^bcd^	2.65	3.43	3.97 ^bc^
24	0	42.7 ^d^	0.719 ^cd^	0.201 ^de^	0.071 ^de^	1.85 ^abcd^	2.60	3.47	3.58 ^de^
	2	46.4 ^d^	0.692 ^fg^	0.216 ^bc^	0.084 ^ab^	1.86 ^abcd^	2.93	3.83	3.21 ^fg^
	4	47.0 ^d^	0.696 ^efg^	0.216 ^bc^	0.078 ^bcde^	1.70 ^bcd^	2.74	3.71	3.22 ^fg^
	6	41.7 ^d^	0.709 ^de^	0.206 ^cd^	0.078 ^bcde^	1.98 ^abcd^	2.80	3.48	3.45 ^ef^
	8	45.9 ^d^	0.702 ^ef^	0.215 ^bc^	0.076 ^bcde^	1.59 ^d^	2.59	3.39	3.27 ^fg^
48	0	54.4 ^c^	0.698 ^efg^	0.217 ^bc^	0.076 ^bcde^	2.14 ^ab^	3.01	4.13	3.24 ^fg^
	2	61.5 ^ab^	0.667 ^h^	0.233 ^a^	0.089 ^a^	2.04 ^abc^	3.66	5.18	2.86 ^h^
	4	62.6 ^a^	0.673 ^h^	0.235 ^a^	0.082 ^abc^	2.19 ^a^	3.18	4.88	2.88 ^h^
	6	56.8 ^bc^	0.691 ^fg^	0.224 ^ab^	0.076 ^bcde^	2.14 ^ab^	2.89	4.27	3.09 ^gh^
	8	54.9 ^c^	0.686 ^g^	0.229 ^a^	0.075 ^bcde^	2.09 ^ab^	2.95	4.33	2.99 ^gh^
SEM		1.10	0.0024	0.0019	0.0009	0.036	0.213	0.276	0.047
*p*-values									
Time		<0.001	<0.001	<0.001	<0.001	<0.001	0.610	0.167	<0.0001
Linear		0.006	<0.001	0.013	<0.001	0.540	0.715	0.976	0.007
Quadratic		0.291	0.591	0.227	0.483	0.645	0.793	0.167	0.436
Time × Trt		<0.001	<0.001	<0.001	<0.001	0.010	1.000	0.989	<0.001

Trt, treatment; TVFA, total volatile fatty acids; A:P Ratio, acetate/propionate ratio. ^a–h^ Means with different superscripts within each column differ by *p* < 0.05.

**Table 5:** Estimated parameters on effect of inoculation period on for treated and untreated corn stover.

Variables	Fermentation Period, wk						Contrasts (*p*-Values)	
	0	2	4	6	8	SEM	Linear	Quadratic
DMI	1.68	2.05	2.07	1.81	1.84	0.035	<0.001	<0.001
TDN	46.8	51.3	50.1	44.4	44.3	0.72	0.001	0.016
DDM	54.1	56.8	56.1	52.7	52.6	0.44	0.001	0.016
RFV	70.5	90.2	90.0	74.1	75.2	2.02	<0.001	0.189
RFQ	64.0	85.5	84.4	65.5	66.4	2.40	<0.001	0.833
DCP	0.044	1.89	1.86	1.43	1.62	0.1664	<0.001	<0.001
GE	4.56	4.28	4.31	4.23	4.39	0.030	<0.001	0.011
DE	2.06	2.26	2.21	1.96	1.95	0.032	0.001	0.016
ME	1.69	1.86	1.81	1.61	1.60	0.026	0.001	0.016
NEM	1.07	1.19	1.16	0.998	0.994	0.0209	0.001	0.016
NEG	0.348	0.478	0.442	0.278	0.274	0.0209	0.001	0.016
NEL	1.14	1.25	1.22	1.08	1.07	0.018	0.001	0.016

DMI, dry matter intake; TDN, total digestible nutrient; DDM, digestible dry matter; RFV, relative forage value; RFQ, relative forage quality; DCP, digestible crude protein; GE, gross energy; DE, digestible energy; ME, metabolizable energy; NEM, net energy of maintenance; NEG, net energy of gain; NEL, net energy of lactation.

## Data Availability

Data sharing not applicable.

## References

[1] Julian A.V., Reyes R.G., Eguchi F. (2019). Agro-Industrial Waste Conversion Into Medicinal Mushroom Cultivation. Encyclopedia of Environmental Health.

[2] Asmare B. (2020). Biological Treatment of Crop Residues as an Option for Feed Improvement in the Tropics: A Review. Anim. Husb. Dairy Vet. Sci..

[3] Kuhad R.C., Kuhar S., Sharma K.K., Shrivastava B., Kuhad R.C., Singh A. (2013). Microorganisms and Enzymes Involved in Lignin Degradation Vis-à-Vis Production of Nutritionally Rich Animal Feed: An Overview. Biotechnology for Environmental Management and Resource Recovery.

[4] Fazaeli H., Masoodi A.R. (2006). Spent Wheat Straw Compost of Agaricus Bisporus Mushroom as Ruminant Feed. Asian-Australas J. Anim. Sci..

[5] Gargano M., Van Griensven L., Isikhuemhen O., Lindequist U., Venturella G., Wasser S., Zervakis G. (2017). Medicinal Mushrooms: Valuable Biological Resources of High Exploitation Potential. Plant Biosyst. Int. J. Deal. All Asp. Plant Biol..

[6] Isikhuemhen O.S., Mikiashvilli N.A. (2009). Lignocellulolytic Enzyme Activity, Substrate Utilization, and Mushroom Yield by Pleurotus Ostreatus Cultivated on Substrate Containing Anaerobic Digester Solids. J. Ind. Microbiol. Biotechnol..

[7] Melanouri E.-M., Dedousi M., Diamantopoulou P. (2022). Cultivating Pleurotus Ostreatus and Pleurotus Eryngii Mushroom Strains on Agro-Industrial Residues in Solid-State Fermentation. Part I: Screening for Growth, Endoglucanase, Laccase and Biomass Production in the Colonization Phase. Carbon Resour. Convers..

[8] Shrivastava B., Thakur S., Khasa Y.P., Gupte A., Puniya A.K., Kuhad R.C. (2011). White-Rot Fungal Conversion of Wheat Straw to Energy Rich Cattle Feed. Biodegradation.

[9] Pandey V.K., Singh M.P. (2014). Biodegradation of Wheat Straw by Pleurotus Ostreatus. Cell Mol. Biol..

[10] Barh A., Kumari B., Sharma S., Annepu S.K., Kumar A., Kamal S., Sharma V.P., Bhatt P. (2019). Chapter 1—Mushroom Mycoremediation: Kinetics and Mechanism. Smart Bioremediation Technologies.

[11] Adesogan A.T., Arriola K.G., Jiang Y., Oyebade A., Paula E.M., Pech-Cervantes A.A., Romero J.J., Ferraretto L.F., Vyas D. (2019). Symposium Review: Technologies for Improving Fiber Utilization. J. Dairy Sci..

[12] van Kuijk S.J.A., Sonnenberg A.S.M., Baars J.J.P., Hendriks W.H., Cone J.W. (2015). Fungal Treatment of Lignocellulosic Biomass: Importance of Fungal Species, Colonization and Time on Chemical Composition and in vitro Rumen Degradability. Anim. Feed Sci. Technol..

[13] Koutrotsios G., Mountzouris K.C., Chatzipavlidis I., Zervakis G.I. (2014). Bioconversion of Lignocellulosic Residues by Agrocybe Cylindracea and Pleurotus Ostreatus Mushroom Fungi—Assessment of Their Effect on the Final Product and Spent Substrate Properties. Food Chem..

[14] Bhandari B. Crop Residue as Animal Feed; European Master in Sustainable Animal Nutrition and Feeding: 2019. https://www.researchgate.net/publication/335490928_CROP_RESIDUE_AS_ANIMAL_FEED.

[15] Soto-SAnchez A., Ramirez-Bribiesca J.E., Meneses-Mayo M., Loera-Corral O., Miranda-Romero L.A., BArcena-Gama R. (2015). Effects of *Pleurotus sapidus* (Schulzer) Sacc. Treatment on Nutrient Composition and Ruminal Fermentability of Barley Straw, Barley Rootless, and a Mixture of the Two. Chil. J. Agric. Res..

[16] Olagunju L.K., Isikhuemhen O.S., Dele P.A., Anike F.N., Essick B.G., Holt N., Udombang N.S., Ike K.A., Shaw Y., Brice R.M. (2023). Pleurotus Ostreatus Can Significantly Improve the Nutritive Value of Lignocellulosic Crop Residues. Agriculture.

[17] Anele U.Y., Refat B., Swift M.-L., He Z.X., Zhao Y.L., McAllister T.A., Yang W.Z. (2014). Effects of Bulk Density, Precision Processing and Processing Index on in vitro Ruminal Fermentation of Dry-Rolled Barley Grain. Anim. Feed Sci. Technol..

[18] McDougall E.I. (1948). Studies on Ruminant Saliva. 1. The Composition and Output of Sheep’s Saliva. Biochem. J..

[19] Mauricio R.M., Mould F.L., Dhanoa M.S., Owen E., Channa K.S., Theodorou M.K. (1999). A Semi-Automated in vitro Gas Production Technique for Ruminant Feedstuff Evaluation. Anim. Feed Sci. Technol..

[20] Thiex N., Novotny L., Crawford A. (2012). Determination of Ash in Animal Feed: AOAC Official Method 942.05 Revisited. J. AOAC Int..

[21] Latimer George W. (2019). Official Methods of Analysis of AOAC International.

[22] Van Soest P.J., van Robertson J.B., Lewis B.A. (1991). Methods for Dietary Fiber, Neutral Detergent Fiber, and Nonstarch Polysaccharides in Relation to Animal Nutrition. J. Dairy Sci..

[23] Robertson J.B., Van Soest P.J., James W.P.T., Theander O., James W.P.T., Theander O. (1981). The Analysis of Dietary Fiber in Food. The Detergent System of Analysis and Its Application to Human Foods.

[24] Blümmel M., Lebzien P. (2001). Predicting Ruminal Microbial Efficiencies of Dairy Rations by in vitro Techniques. Livest. Prod. Sci..

[25] Blümmel M., Becker K. (1997). The Degradability Characteristics of Fifty-Four Roughages and Roughage Neutral-Detergent Fibres as Described by in vitro Gas Production and Their Relationship to Voluntary Feed Intake. Br. J. Nutr..

[26] Siqueira M., Chagas J., Monnerat J.P., Monteiro C., Mora-Luna R., Dubeux J., Dilorenzo N., Ruiz-Moreno M., Ferreira M. (2021). Nutritive Value, in vitro Fermentation, and Methane Production of Cactus Cladodes, Sugarcane Bagasse, and Urea. Animals.

[27] Ali H.A.M., Ismail A.B.O., Fatur M., Ahmed F.A., Ahmed E.H.O., Ahmed M.E.E. (2016). Nutritional Evaluation and Palatability of Major Range Forbs from South Darfur, Sudan. Open J. Anim. Sci..

[28] Hall J.A., Melendez L.D., Jewell D.E. (2013). Using Gross Energy Improves Metabolizable Energy Predictive Equations for Pet Foods Whereas Undigested Protein and Fiber Content Predict Stool Quality. PLoS ONE.

[29] Jeranyama P., Garcia A.D. (2004). Understanding Relative Feed Value (RFV) and Relative Forage Quality (RFQ).

[30] Mudau H.S., Mokoboki H.K., Ravhuhali K.E., Mkhize Z. (2021). Nutrients Profile of 52 Browse Species Found in Semi-arid Areas of South Africa for Livestock Production: Effect of Harvesting Site. Plants.

[31] O’shea J., Maguire M.F. (1967). Relationship between Crude Protein and Digestible Crude Protein Content of Feedstuffs for Ruminants. Ir. J. Agric. Res..

[32] SAS Institute Inc (2014). Statistical Analysis Software SAS/STAT®.

[33] Sánchez C. (2009). Lignocellulosic Residues: Biodegradation and Bioconversion by Fungi. Biotechnol. Adv..

[34] Hoffman P.C. (2005). Ash Content of Forages. Focus Forage.

[35] Khattab H.M., Gado H., Salem A.Z.M., Diaz L.M., El-Sayed M., Kholif A., Elshewy A., Kholif A. (2013). Chemical Composition and In vitro Digestibility of Pleurotus Ostreatus Spent Rice Straw. Anim. Nutr. Feed Technol..

[36] Nayan N., Sonnenberg A.S.M., Hendriks W.H., Cone J.W. (2018). Variation in the Solubilization of Crude Protein in Wheat Straw by Different White-Rot Fungi. Anim. Feed Sci. Technol..

[37] Nayan N., Sonnenberg A.S.M., Heniks W.H., Cone J.W. (2018). Screening of White-Rot Fungi for Bioprocessing of Wheat Straw into Ruminant Feed. J. Appl. Microbiol..

[38] Atalar A., Çetinkaya N. (2020). Treatment of Corn Straw with Pleurotus Ostreatus, Pleurotus Eryngii and Lentinula Edodes to Improve the Digestibility of the Lignocellulosic Complex. JOR.

[39] Schneider I., Kressel G., Meyer A., Krings U., Berger R.G., Hahn A. (2011). Lipid Lowering Effects of Oyster Mushroom (*Pleurotus ostreatus*) in Humans. J. Funct. Foods..

[40] Nurfitri N., Mangunwardoyo W., Saskiawan I. (2021). Lignocellulolytic Enzyme Activity Pattern of Three White Oyster Mushroom (*Pleurotus ostreatus* (Jacq.) P. Kumm.) Strains during Mycelial Growth and Fruiting Body Development. J. Phys. Conf. Ser..

[41] Brice R.M., Dele P.A., Ike K.A., Shaw Y.A., Olagunju L.K., Orimaye O.E., Subedi K., Anele U.Y. (2022). Effects of Essential Oil Blends on In vitro Apparent and Truly Degradable Dry Matter, Efficiency of Microbial Production, Total Short-Chain Fatty Acids and Greenhouse Gas Emissions of Two Dairy Cow Diets. Animals.

[42] Chanjula P., So S., Suntara C., Prachumchai R., Cherdthong A. (2022). Efficiency of Feed Utilization, Ruminal Traits, and Blood Parameters of Goats Given a Total Mixed Diet Ration Containing Extracted Oil Palm Meal. Vet. Sci..

[43] Anike F.N., Yusuf M., Isikhuemhen O.S. (2016). Co-Substrating of Peanut Shells with Cornstalks Enhances Biodegradation by Pleurotus Ostreatus. J. Bioremediation Biodegredation.

[44] Zadrazil F. (1997). Changes in In vitro Digestibility of Wheat Straw During Fungal Growth and after Harvest of Oyster Mushrooms (*Pleurotus* Spp.) on Laboratory and Industrial Scale. J. Appl. Anim. Res..

[45] Nayan N., Sonnenberg A.S.M., Hendriks W.H., Cone J.W. (2020). Prospects and Feasibility of Fungal Pretreatment of Agricultural Biomass for Ruminant Feeding. Anim. Feed Sci. Technol..

[46] Adamović M., Grubić G., Ivanka M., Jovanović R., Protić R., Ljiljana S., Lj S. (1998). The Biodegradation of Wheat Straw by Pleurotus Ostreatus Mushrooms and Its Use in Cattle Feeding. Anim. Feed Sci. Technol..

[47] Tuyen D.V., Phuong H.N., Cone J.W., Baars J.J.P., Sonnenberg A.S.M., Hendriks W.H. (2013). Effect of Fungal Treatments of Fibrous Agricultural By-Products on Chemical Composition and in vitro Rumen Fermentation and Methane Production. Bioresour. Technol..

[48] Naidu Y., Siddiqui Y., Idris A.S. (2020). Comprehensive Studies on Optimization of Ligno-Hemicellulolytic Enzymes by Indigenous White Rot Hymenomycetes under Solid-State Cultivation Using Agro-Industrial Wastes. J. Environ. Manag..

[49] Islamiyati R., Rasjid S., Natsir A. (2013). Ismartoyo Crude Protein And Fiber Fraction Of Corn Stover Inoculated By Fungi *Trichoderma* Sp. and *Phanerochaete chrysosporium*. Int. J. Sci. Technol. Res..

[50] Mk G., Krishna C., Moo-Young M. (2001). Fungal Solid State Fermentation—An Overview. Appl. Mycol. Biotechnol..

[51] Lechner B.E., Papinutti V.L. (2006). Production of Lignocellulosic Enzymes during Growth and Fruiting of the Edible Fungus Lentinus Tigrinus on Wheat Straw. Process Biochem..

[52] Melanouri E.-M., Dedousi M., Diamantopoulou P. (2022). Cultivating Pleurotus Ostreatus and Pleurotus Eryngii Mushroom Strains on Agro-Industrial Residues in Solid-State Fermentation. Part II: Effect on Productivity and Quality of Carposomes. Carbon Resour. Convers..

[53] Bowen M.K., Poppi D.P., McLennan S.R. (2017). Efficiency of Rumen Microbial Protein Synthesis in Cattle Grazing Tropical Pastures as Estimated by a Novel Technique. Anim. Prod. Sci..

[54] Blümmel M., Steingaβ H., Becker K. (1997). The Relationship between in vitro Gas Production, in vitro Microbial Biomass Yield and 15N Incorporation and Its Implications for the Prediction of Voluntary Feed Intake of Roughages. Br. J. Nutr..

[55] Katoch R., Apoorva A., Tripathi S. (2017). Sood improving nutritive value and digestibility of maize stover—A review. Forage Res..

[56] Fazaeli H., Mahmodzadeh H., Azizi A., Jelan Z.A., Liang J.B., Rouzbehan Y., Osman A. (2004). Nutritive Value of Wheat Straw Treated with Pleurotus Fungi. Asian-Australas J. Anim. Sci..

[57] Weiss W.P., Tebbe A.W. (2019). Estimating Digestible Energy Values of Feeds and Diets and Integrating Those Values into Net Energy Systems. Transl. Anim. Sci..

